# Benchmarking the scientific output of industrial wastewater research in Arab world by utilizing bibliometric techniques

**DOI:** 10.1007/s11356-016-6434-6

**Published:** 2016-03-21

**Authors:** Shaher H. Zyoud, Aiman E. Al-Rawajfeh, Hafez Q. Shaheen, Daniela Fuchs-Hanusch

**Affiliations:** Institute of Urban Water Management and Landscape Water Engineering, Graz University of Technology, Stremayrgasse 10/I, A-8010 Graz, Austria; Chemical Engineering Department, The University of Jordan, Amman, Jordan; Chemical Engineering Department, Tafila Technical University, Tafila, Jordan; Civil Engineering Department, An-Najah National University, Nablus, Palestine

**Keywords:** Arab world, Benchmarking, Bibliometrics, Industrial wastewater, Scopus, *h*-index

## Abstract

Rapid population growth, worsening of the climate, and severity of freshwater scarcity are global challenges. In Arab world countries, where water resources are becoming increasingly scarce, the recycling of industrial wastewater could improve the efficiency of freshwater use. The benchmarking of scientific output of industrial wastewater research in the Arab world is an initiative that could support in shaping up and improving future research activities. This study assesses the scientific output of industrial wastewater research in the Arab world. A total of 2032 documents related to industrial wastewater were retrieved from 152 journals indexed in the Scopus databases; this represents 3.6 % of the global research output. The *h*-index of the retrieved documents was 70. The total number of citations, at the time of data analysis, was 34,296 with an average citation of 16.88 per document. Egypt, with a total publications of 655 (32.2 %), was ranked the first among the Arab countries followed by Saudi Arabia 300 (14.7 %) and Tunisia 297 (14.6 %). Egypt also had the highest *h*-index, assumed with Saudi Arabia, the first place in collaboration with other countries. Seven hundred fifteen (35.2 %) documents with 66 countries in Arab/non-Arab country collaborations were identified. Arab researchers collaborated mostly with researchers from France 239 (11.7 %), followed by the USA 127 (6.2 %). The top active journal was *Desalination* 126 (6.2 %), and the most productive institution was the National Research Center, Egypt 169 (8.3 %), followed by the King Abdul-Aziz University, Saudi Arabia 75 (3.7 %). Environmental Science was the most prevalent field of interest 930 (45.8 %). Despite the promising indicators, there is a need to close the gap in research between the Arab world and the other nations. Optimizing the investments and developing regional experiences are key factors to promote the scientific research.

## Introduction

With continuing urbanization and industrialization across the globe, associated with increase in population, the pollution of aquatic environments caused by industrial wastewaters has increased and became a major environmental concern (Azizullah et al. 2015). Industrial wastewater acts as a major source of toxic organic compounds (Wu et al. 2015) and heavy metals to natural waters (Abramov et al. 2014), which are of concern for public health (Phetphaisit et al. 2016). The uncontrolled disposal of industrial wastewaters often results in contamination of surface and groundwater and imbalance of ecosystems. In the developing world, 70 % of untreated industrial wastewaters are released into surface water (Azizullah et al. 2015).

Major sources of industrial wastewater include iron and steel industry, mining, food industry, nuclear industry, complex organic chemical industry, textiles and leather, pulp and paper, microelectronics industry, and pharmaceutical industry (Lin et al. 2012). The characteristics of industrial wastewaters depend substantially on the industrial processes and the produced wastewater. These characteristics vary within each industry and diverse than the municipal/domestic wastewaters which have similar composition in terms of quality and quantity (Krzemińska et al. 2015).

The large variations in the composition and loads of industrial wastewaters, the presence of high concentrations of salts and organic matters, and the poorly biodegradable organic substances hinder treating these effectively (Dvořák et al. 2013; García-García et al. 2015; Szafnicki and Narce 2006). Therefore, industries and researchers devoted their efforts to develop technologies and processes characterized by higher efficiencies and inexpensive costs aiming to reduce the volumes of wastewater at source, to improve the quality of the effluents (Barakat 2011), and to meet the allowable maximum contaminant levels and standards.

The most prevalent techniques in industrial wastewater treatment realm comprise (a) adsorption which is a conventional method and a highly effective technique in removing heavy metals (Oke et al. 2008); (b) membrane separation which has been used recently for the treatment of inorganic effluent due to its convenient operation (Barakat 2011); (c) electro treatments such as electrodialysis, which is friendly for environment (Barakat 2011; Pedersen 2003); (d) photocatalytic which is an innovative and promising technique for efficient destruction of pollutants (Barakat 2011); (e) integration of electro-oxidation and ozonation which is an advance technique to reduce the high organic load of industrial wastewater (García-Morales et al. 2013); (f) electrochemical advanced oxidation which is efficient in removing pharmaceuticals from water (Brillas and Sirés 2015); and (g) nanotechnology technique which is advancing (Roy and Bhattacharya 2015).

Up to date, the available treatment technologies experience series of technical and economic challenges with regard to developing effective techniques to reclaim the industrial effluents. This has led to extensive research to overcome the inherent limitations in the existing technologies (Roy and Bhattacharya 2015).

## Industrial wastewater research in the Arab world

As water scarcity becomes a more pressing concern throughout the Arab world, water reuse is considered as a great potential in increasing water resources (World Bank 2011). The estimated amount of wastewater produced by the Arab world is 10.8 km^3^/year (World Bank 2011). Researchers and policy makers are searching continuously for additional water sources. Reuse-treated wastewater and recycling the industrial wastewater in industrial sectors are now considered as supplementary sources with significant potential in alleviating water scarcity (Al-Zubari 1998).

Geographically, the Arab world lies between the Atlantic coasts of northern Africa and the Arabian Gulf (Saleh 2015). The 22 Arab countries, with approximately 370 million individuals, comprise a region with great diversity in socioeconomic conditions, but there are important commonalities in language, culture, religion, and demographics (Obermeyer et al. 2015). Since two decades, the scientific research in the Arab region has shown a general trend of growth (Waast and Rossi 2010) and the research productivity has increased from 0.6 % out of global productivity in 1981 to 0.9 % in 2000 (UIS 2005). The recent development in living standards, educational levels, and health services in most of Arab countries has been associated with the progress in scientific research (Benamer and Bakoush 2009). The negligence of science in this region which has a historical and catalytic role in the scientific revolution in its early stages (Maziak 2005) and a proud history of scholarship is dwindling, and grassroot initiatives with a hope in restoring the balance are witnessed (Masood 2002). The spending on research and development is on the rise (Masood 2002).

The assessment of the Arab world’s contribution in industrial wastewater research through mapping the productivity of research will help in identifying the existing research directions and the state of research with comparison to other nations. It will assist future research investments since the advances in sciences and technologies are becoming rapidly important for regional competitiveness and economic growth (Minguillo et al. 2015). Bibliometric techniques, which mainly utilize quantitative analysis and statistical indices to evaluate research productivity of individuals, institutes, or countries, are valuable tools in measuring the scientific research productivity (Wallin 2005). These tools can be used to make pronouncements about qualitative pictures of scientific activities (Wallin 2005). A good knowledge and information related to the state of research in a particular discipline, which could assist the researchers to identify and conduct new lines of research, can be derived from the output measurements of the bibliometric techniques (De Battisti and Salini 2013). These techniques are well-known research procedures with high potential to perform systematic analyses (van Raan 2005).

By reviewing the available literature, and up to the authors’ knowledge, the evaluation of research productivity related to industrial wastewater research in the Arab world has not been addressed. Globally, many studies have tackled this issue including Zheng et al. (2015) and Qian et al. (2015), respectively. The first has evaluated industrial wastewater treatment research from 1991 to 2014 based on data from the Science Citation Index Expanded database (Zheng et al. 2015). The second study has evaluated the research patterns and tendencies of pharmaceutical wastewater treatment from 1994 to 2013 based on data from the Science Citation Index Expanded database and Web of Science (Qian et al. 2015).

Our main objective is to analyze the research productivity of the Arab world in industrial wastewater. This analysis will lead to better understanding of the current status of industrial wastewater research in the Arab world. It aims in helping researchers and policy makers in shaping future research activities.

## Methodology

The data used in this study was harvested from Scopus databases. These databases are considered one of the largest databases of peer-reviewed literature, include more than 57 million records, and cover over 21,000 peer-reviewed journals (Elsevier 2015). All Arab countries within Arab league, Saudi Arabia, Egypt, Jordan, Palestine, Lebanon, Qatar, Bahrain, Kuwait, Morocco, Tunisia, Syrian Arab Republic, United Arab Emirates, Iraq, Sudan, Yemen, Algeria, Comoros, Djibouti, Libya, Mauritania, Oman, and Somalia, are used as country keys in this study. The search covered all subject areas within Scopus that comprise health, social, life, and physical sciences. The output of scientific research after the year 2014 has been excluded because the period after December 31, 2013 is still open for new publications. The search was implemented one time on October 5, 2015 to eliminate the bias which may appear because of continuous updating of Scopus databases.

The search expression used in the advanced search is the same used in the study, which has been conducted by Zheng et al. (2015), to evaluate the scientific research productivity in industrial wastewater treatment. We kept the lower boundary of the search period as an open interval, and the upper is the end of the year 2013. The analysis was limited to articles and review articles and has excluded all other types of publications such as books and conference papers. The topic search contains the fields of each paper’s title and abstract. The search query appeared like the following pattern in the advanced search: TITLE OR ABS (industrial OR industry) AND (sewage* OR effluent* OR wastewater* OR (waste water*) OR waste-water*) AND PUBYEAR < 2014 AND (EXCLUDE (DOCTYPE, “cp”) OR EXCLUDE (DOCTYPE, “ch”) OR EXCLUDE (DOCTYPE, “bk”) OR EXCLUDE (DOCTYPE, “cr”) OR EXCLUDE (DOCTYPE, “no”) OR EXCLUDE (DOCTYPE, “sh”) OR EXCLUDE (DOCTYPE, “bz”) OR EXCLUDE (DOCTYPE, “rp”) OR EXCLUDE (DOCTYPE, “ed”) OR EXCLUDE (DOCTYPE, “ab”) OR EXCLUDE (DOCTYPE, “le”) OR EXCLUDE (DOCTYPE, “er”) OR EXCLUDE (DOCTYPE, “Undefined”)) AND (LIMIT-TO (AFFILCOUNTRY, “Egypt”) OR LIMIT-TO (AFFILCOUNTRY, “Saudi Arabia”) OR LIMIT-TO (AFFILCOUNTRY, “Tunisia”) OR LIMIT-TO (AFFILCOUNTRY, “Algeria”) OR LIMIT-TO (AFFILCOUNTRY, “Morocco”) OR LIMIT-TO (AFFILCOUNTRY, “Jordan”) OR LIMIT-TO (AFFILCOUNTRY, “United Arab Emirates”) OR LIMIT-TO (AFFILCOUNTRY, “Kuwait”) OR LIMIT-TO (AFFILCOUNTRY, “Oman”) OR LIMIT-TO (AFFILCOUNTRY, “Lebanon”) OR LIMIT-TO (AFFILCOUNTRY, “Qatar”) OR LIMIT-TO (AFFILCOUNTRY, “Iraq”) OR LIMIT-TO (AFFILCOUNTRY, “Palestine”) OR LIMIT-TO (AFFILCOUNTRY, “Syrian Arab Republic”) OR LIMIT-TO (AFFILCOUNTRY, “Libyan Arab Jamahiriya”) OR LIMIT-TO (AFFILCOUNTRY, “Bahrain”) OR LIMIT-TO (AFFILCOUNTRY, “Yemen”) OR LIMIT-TO (AFFILCOUNTRY, “Sudan”)).

Through analyzing and auditing the list of countries from the global, four Arab countries did not have any contribution toward research articles related to industrial wastewater. They are Comoros, Djibouti, Mauritania, and Somalia. The output data have been analyzed to create a perspective about (a) research productivity in industrial wastewater research, (b) collaboration patterns between the Arab countries and the world, (c) citations of the published research, (d) journals in which researchers from Arab world have published their works, and (e) comparative study with other nations in the same region (Turkey, Israel, and Iran), which was done to measure the performance of Arab countries in industrial wastewater research as the base in benchmarking analysis.

The top ten ranked outputs from the bibliometric measurements (countries, institutions, areas of interests, and cited articles) are displayed. They attract ranking in order of descending according to the formula of standard competition ranking (SCR). In case two measurements attracted the same ranking, a gap has to be considered for the following numbers.

The *h*-index, which represents an indicator with a potential to incorporate both quantity (publication) and quality (citation scores) measures (Egghe 2006), has been extracted to demonstrate the total citations of the published works. This index was proposed to characterize the importance, significance, and broad impact of a researcher’s cumulative research contributions (Hirsch 2005) and to qualify the performance of research (Meho and Rogers 2008). To illustrate its concept on testing a country’s scientific impact and productivity, a country with *h*-index 30 has published 30 documents and each document has gained at least 30 citations. Journal Citation Report (JCR; Web of Knowledge 2014) has been employed to display the impact factor (IF) of the journals and considered top ten ranked journals.

The Statistical Package for Social Sciences (SPSS) program version 20 has been used to calculate statistic measurements, which comprise descriptive statistic components, such as median, mean, and sum, and the median (Q1–Q3 interquartile range).

## Results

At global level, the total number of retrieved documents from Scopus databases was 78,836 documents and the exclusion of all types of publications except the articles and review articles lead to a total of 57,235 documents. By limiting the search to articles and review articles that have been published by researchers from the Arab world, the total output was 2032 documents. This figure represents 3.6 % of the global research productivity in the fields of industrial wastewater.

**Fig. 1:**
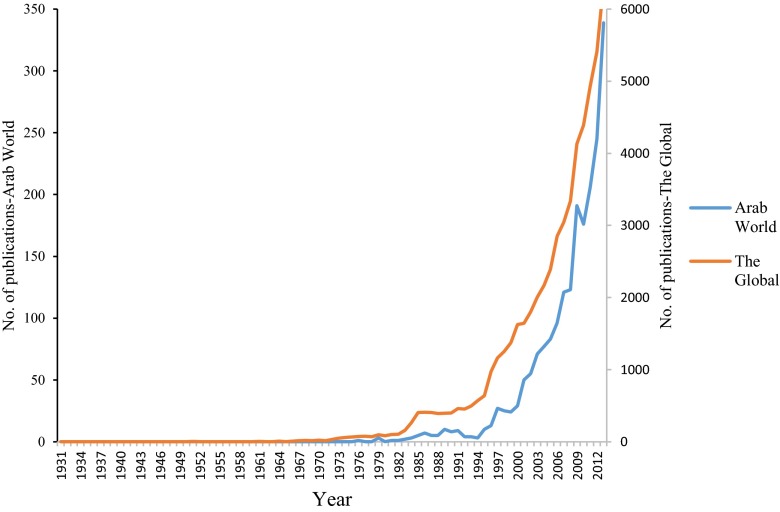
Number of published articles and review articles from the Arab world and global in industrial wastewater research

The industrial wastewater research activities by researchers in the Arab world have begun in 1976. The productivity grew at a very modest rate and breakthrough has occurred in 2001, as shown in Fig. 1. More than 90 % of the articles were published after the year 2001. The first published article was in the *International Journal of Mineral Processing* by Doheim, M.A. from the Faculty of Engineering, Assiut University, Egypt, entitled “Fluidization in the non-ferrous mineral processing and metal industry” (Doheim 1976). At global level, the first published work as documented in Scopus databases was in 1931 in the *Journal of Chemical Education* by Arnold, L.K. from Engineering Experiment Station, Iowa State College, USA, entitled “Agricultural wastes in industry” (Arnold 1931).

The analysis of the used language in the published works shows that the English language is predominant (1957; 96.3 %), followed in far away by French (80; 3.9 %), Spanish (6; 0.3 %), Italian (2; 0.1 %), and Korean (1; 0.05 %).

The analyzing of data for countries of the Arab world (Table 1) shows that 18 countries out of 22 of the Arab world league have contributions in industrial wastewater research. Their contributions and collaboration patterns with other countries and citation rates vary from one country to another. Egypt had the highest research output (655; 32.23 %), followed by Saudi Arabia (300; 14.76 %), Tunisia (297; 14.62 %), and Algeria (200; 9.84 %). The total number of citations at the time of processing the analysis (October 5, 2015) was 34,296, with a mean of 16.88 and a median (interquartile range) of 6 (1.0–16.0). At country level, Egypt had the highest number of citations (8044), followed by Tunisia (6171), Saudi Arabia (5104), and Morocco (4249). The *h*-index of the total retrieved documents was 70, which means that 70 documents had attracted at least 70 citations to the time of data analysis. The highest *h*-index was 43 for Egypt, followed by 36 for Saudi Arabia, and 35 for Tunisia.

**Table 1:** Bibliometric analysis of the 2032 documents associated with industrial wastewater research output by Arab countries

SCR^a^	Countries	Articles (%)	*h*-index	No. of citations	Median citation (Q1–Q3)	Average citation	Collaborations with other countries	Number (%)^b^ of documents with international authors	Most collaborated country	No. of documents with most collaborated country (%)
1st	Egypt	655 (32.23)	43	8044	4 (1.0–14.0)	12.28	42	183 (27.9)	Saudi Arabia	55 (8.4)
2nd	Saudi Arabia	300 (14.76)	36	5104	6.5 (2.0–16.0)	17.01	42	179 (59.7)	Egypt	55 (18.3)
3rd	Tunisia	297 (14.62)	35	6171	9 (3.0–20.0)	20.78	31	139 (46.8)	France	78 (26.3)
4th	Algeria	200 (9.84)	23	2240	4 (1.0–14.75)	11.2	20	95 (47.5)	France	77 (38.5)
5th	Morocco	172 (8.46)	27	4249	5.5 (1.0–17.0)	24.7	22	101 (58.7)	France	63 (36.6)
6th	Jordan	133 (6.55)	22	1790	6 (1.5–18.0)	13.46	29	51 (38.3)	USA	12 (9.0)
7th	United Arab Emirates	96 (4.72)	21	4120	8.5 (3.0–18.0)	42.92	22	59 (61.5)	USA	10 (10.4)
8th	Kuwait	74 (3.64)	16	873	3 (1.0–14.25)	11.8	21	23 (31.1)	Canada	5 (6.8)
9th	Oman	41 (2.02)	13	603	7 (2.0–18.5)	14.71	24	27 (65.9)	USA	5 (12.2)
10th	Lebanon	39 (1.92)	13	621	9 (3.0–18.0)	15.92	12	27 (69.2)	France	12 (30.8)
11th	Qatar	38 (1.87)	17	634	9 (1.0–26.5)	16.68	21	31 (81.6)	Egypt	8 (21.1)
12th	Iraq	35 (1.72)	8	221	4 (0.0–5.0)	6.31	9	13 (37.1)	Malaysia	3 (8.6)
13th	Palestine	32 (1.57)	10	372	4 (2.0–13.0)	11.63	12	19 (59.4)	USA	6 (18.8)
14th	Syria Arab Republic	26 (1.28)	13	729	11 (3.75–29.25)	28.04	16	14 (53.8)	Sri Lanka	6 (23.1)
15th	Libyan Arab Jamahiriya	17 (0.84)	6	88	3 (0.5–7.0)	5.17	8	8 (47.1)	Egypt	2 (11.8)
15th	Bahrain	17 (0.84)	10	292	11 (1.5–20.0)	17.18	15	5 (29.4)	France	2 (11.8)
17th	Yemen	9 (0.44)	4	271	4 (1.0–10.5)	30.11	9	6 (66.7)	Iraq	2 (22.2)
18th	Sudan	8 (0.39)	4	89	3 (1.25–12.75)	11.13	19	5 (62.5)	India	2 (25.0)
19th	Mauritania	–	–	–	–	–	–	–	–	–
19th	Djibouti	–	–	–	–	–	–	–	–	–
19th	Somalia	–	–	–	–	–	–	–	–	–
19th	Comoros	–	–	–	–	–	–	–	–	–

*SCR* standard competition ranking, *Q1–Q3* lower quartile–upper quartile

^a^Equal countries have the same ranking number, and then, a gap is left in the ranking numbers

^b^Percentage of documents with international authors (i.e., from other Arab and non-Arab countries) from the total number of documents for each country

Table 2 lists the regions and countries of the world whose researchers have collaborations with researchers from the Arab world in industrial wastewater research. The study identified 715 (35.19 %) documents with 66 countries in the Arab world collaboration with non-Arab countries. At regional level, Arab researchers collaborated mostly with researchers from Western Europe (423; 20.82 %), followed by researchers from Northern America (166; 8.17 %) and Asiatic region (152; 7.48 %). At county level, France recorded the high rate of collaboration with the Arab world (*n* = 239), followed by the USA (*n* = 127), UK (*n* = 57), and India (*n* = 52). The highest *h*-index for the published works in the Arab world collaboration with non-Arab countries was 45 from collaboration with Western Europe, followed by 32 with Northern America, and 31 from Asiatic region.

**Table 2:** Collaboration between Arab countries and non-Arab countries in industrial wastewater research

Region/country^a^	No. of documents (%)	Region/country	No. of documents (%)
Western Europe	423 (20.82)^b^	Latin America	21 (1.03)^b^
France	239 (11.71)	Mexico	14 (0.69)
UK	57 (2.81)	Brazil	3 (0.15)
Spain	47 (2.31)	Haiti	1 (0.05)
Germany	44 (2.17)	Guadeloupe	1 (0.05)
Italy	25 (1.23)	Ecuador	1 (0.05)
Netherlands	11 (0.54)	Colombia	1 (0.05)
Belgium	11 (0.54)	Argentina	1 (0.05)
Switzerland	10 (0.49)	Panama	1 (0.05)
Sweden	8 (0.39)	Pacific region	18 (0.89)^b^
Greece	6 (0.3)	Australia	14 (0.69)
Portugal	6 (0.3)	New Zealand	5 (0.25)
Denmark	5 (0.25)	Eastern Europe	18 (0.89)^b^
Austria	4 (0.2)	Russia Federation	6 (0.3)
Ireland	4 (0.2)	Poland	4 (0.2)
Finland	1 (0.05)	Hungary	2 (0.1)
Malta	1 (0.05)	Slovakia	2 (0.1)
North America	166 (8.17)^b^	Czech Republic	1 (0.05)
USA	127 (6.25)	Bulgaria	1 (0.05)
Canada	49 (2.41)	Bosnia and Herzegovina	1 (0.05)
Asiatic region	152 (7.48)^b^	Ukraine	1 (0.05)
India	52 (2.56)	Serbia	1 (0.05)
Malaysia	29 (1.43)	Africa	15 (0.74)^b^
China	28 (1.38)	South Africa	3 (0.15)
Japan	19 (0.94)	Côte d’Ivoire	2 (0.1)
South Korea	16 (0.79)	Ghana	2 (0.1)
Pakistan	9 (0.44)	Kenya	2 (0.1)
Hong Kong	6 (0.30)	Nigeria	2 (0.1)
Sri Lanka	6 (0.3)	Ethiopia	1 (0.05)
Thailand	3 (0.15)	Burkina Faso	1 (0.05)
Singapore	2 (0.1)	Senegal	1 (0.05)
Taiwan	2 (0.1)	Tanzania	1 (0.05)
Bangladesh	2 (0.1)	Middle East	13 (0.64)^b^
Vietnam	2 (0.1)	Turkey	6 (0.3)
Indonesia	1 (0.05)	Iran	4 (0.2)
Laos	1 (0.05)	Israel	3 (0.15)
Kazakhstan	1 (0.05)	Arab-Arab	157 (7.7)
Uzbekistan	1 (0.05)	Arab-Arab	157 (7.7)

^a^The study identified 715 (35.19 %) documents with 66 countries in Arab/non-Arab country collaborations

^b^Total for all regions exceeds 35.19 % as data are overlapping due to multi-country collaboration

Table 3 displays the results of research areas of interests in which Environmental Science was the highest (930; 45.8 %), followed by Chemical Engineering (465; 22.9 %), and Chemistry (380; 18.7 %). The retrieved documents were published in 152 peer-reviewed journals registered in Scopus. Table 4 shows the ranking of the top ten journals where authors from the Arab world have published their works. There were 126 (6.2 %) documents published in the *Desalination* journal, 60 (3 %) in the *Desalination and Water Treatment* journal, and 55 (2.7 %) in the *Journal of Hazardous Materials* journal. Most of the journals in the list of top ten ranking journals (nine journals out of ten) had impact factors as pointed out in JCR 2014.

**Table 3:** Ranking of areas of interests of the published research in the field of industrial wastewater within the period of the study

SCR^a^	Areas of interests	Number (%)^b^
1st	Environmental Science	930 (45.8)
2nd	Chemical Engineering	465 (22.9)
3rd	Chemistry	380 (18.7)
4th	Engineering	333 (16.4)
5th	Agricultural and Biological Sciences	292 (14.4)
6th	Materials Science	231 (11.4)
7th	Biochemistry, Genetics, and Molecular Biology	222 (10.9)
8th	Earth and Planetary Sciences	219 (10.8)
9th	Energy	117 (5.8)
10th	Immunology and Microbiology	115 (5.7)

*SCR* standard competition ranking

^a^Equal areas of interests have the same ranking number, and then, a gap is left in the ranking numbers

^b^Total exceeds 100 % as data are overlapping due to multi-discipline interaction

**Table 4:** Ranking of top 10 journals in which industrial wastewater-related articles were published

SCR^a^	Journal	Frequency	IF (2014)^b^
1st	Desalination	126 (6.2)	3.756
2nd	Desalination and Water Treatment	60 (3.0)	1.173
3rd	Journal of Hazardous Materials	55 (2.7)	4.529
4th	Journal of Chemical Technology and Biotechnology	37 (1.8)	2.349
5th	Chemical Engineering Journal	25 (1.2)	4.321
6th	Environmental Technology	21 (1.0)	1.56
6th	Revue Des Sciences De L Eau	21 (1.0)	NA
8th	Water Research	20 (1.0)	5.528
9th	Environmental Monitoring and Assessment	18 (0.9)	1.679
10th	Environmental Science and Pollution Research	17 (0.8)	2.828

*SCR* standard competition ranking, *NA* not available, *IF* impact factor

^a^Equal journals have the same ranking number, and then, a gap is left in the ranking numbers

^b^The impact factor was reported according to Institute for Scientific Information (ISI) Journal Citation Report (JCR) 2014

The list of the top ten most cited articles is listed in Table 5 (Amine et al. 2006; Bakkali et al. 2008; Banat 1995; Banat et al. 1996; Desai and Banat 1997; Fakhru’l-Razi et al. 2009; Gupta et al. 2010; Houas et al. 2001; Lachheb et al. 2002; Nasef and Hegazy 2004). The most cited review article (1493 citations at the date of analyzing data) was published in the *Food and Chemical Toxicology* journal, followed by articles published in the *Microbiology and Molecular Biology Reviews* journal (1135 citations), the *Bioresource Technology* journal (1013 citations), and the *Applied Catalysis B: Environmental* journal (831). Table 6 lists the top ten most productive institutions and organizations in the Arab world. The most productive institution was the *National Research Centre*, Egypt; followed by *King Abdulaziz University*, Saudi Arabia; and *University of Sfax*, Tunisia.

**Table 5:** Ranking of top ten cited articles in Scopus in the field of industrial wastewater research

SCR^a^	Name of authors and year of publication	Title	Type of document	Journal name	Times cited
1st	Bakkali, F. et al. 2008	Biological effects of essential oils—a review	Review	Food and Chemical Toxicology	1493
2nd	Desai, J.D. and Banat, I.M. 1997	Microbial production of surfactants and their commercial potential	Review	Microbiology and Molecular Biology Reviews	1135
3rd	Banat, I.M. et al. 1996	Microbial decolorization of textile-dye-containing effluents: a review	Article	Bioresource Technology	1013
4th	Houas, A. et al. 2001	Photocatalytic degradation pathway of methylene blue in water	Article	Applied Catalysis B: Environmental	831
5th	Lachheb, H. et al. 2002	Photocatalytic degradation of various types of dyes (alizarin S, crocein orange G, methyl red, Congo red, and methylene blue) in water by UV-irradiated titania	Article	Applied Catalysis B: Environmental	677
6th	Banat, I.M. 1995	Biosurfactants production and possible uses in microbial enhanced oil recovery and oil pollution remediation: a review	Review	Bioresource Technology	352
7th	Nasef, M.M., Hegazy, E.-S.A. 2004	Preparation and applications of ion exchange membranes by radiation-induced graft copolymerization of polar monomers onto non-polar films	Review	Progress in Polymer Science (Oxford)	341
8th	Gupta, V.K. et al. 2010	Adsorption studies on the removal of hexavalent chromium from aqueous solution using a low-cost fertilizer industry waste material	Article	Journal of Colloid and Interface Science	264
9th	Fakhru’l-Razi, A. et al. 2009	Review of technologies for oil- and gas-produced water treatment	Review	Journal of Hazardous Materials	263
10th	Amine, A. et al. 2006	Enzyme inhibition-based biosensors for food safety and environmental monitoring	Review	Biosensors and Bioelectronics	253

*SCR* standard competition ranking

^a^Equal articles have the same ranking number, and then, a gap is left in the ranking numbers

**Table 6:** Ranking of the top ten highly productive institutions in the field of industrial wastewater research during the period of the study

SCR^a^	Name of institution	Country	No. of documents (%)
1st	National Research Centre	Egypt	169 (8.32)
2nd	King Abdulaziz University	Saudi Arabia	75 (3.69)
3rd	University of Sfax	Tunisia	72 (3.54)
4th	Ain Shams University	Egypt	52 (2.56)
5th	Alexandria University	Egypt	48 (2.36)
6th	King Saud University	Saudi Arabia	46 (2.26)
7th	King Fahd University of Petroleum and Minerals	Saudi Arabia	42 (2.07)
8h	Kuwait Institute for Scientific Research	Kuwait	40 (1.97)
9th	United Arab Emirates University	United Arab Emirates	37 (1.82)
9th	Ecole Nationale d’Ingenieurs de Sfax	Tunisia	37 (1.82)

*SCR* standard competition ranking

^a^Equal institutions have the same ranking number, and then, a gap is left in the ranking numbers

**Fig. 2:**
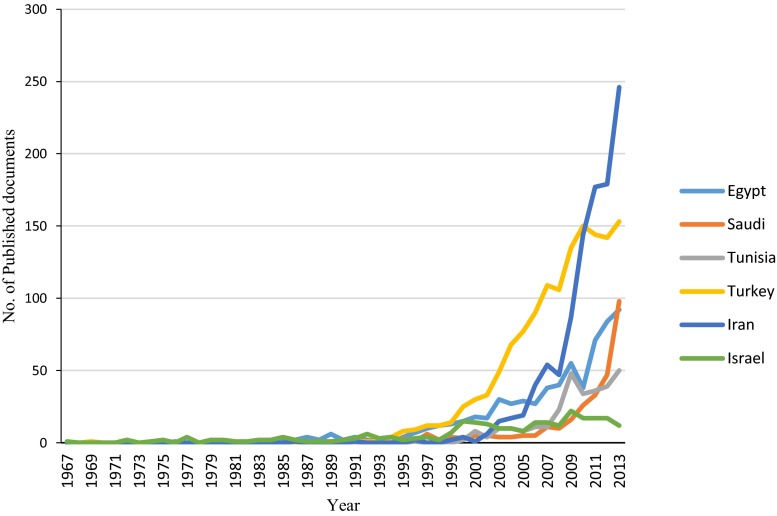
Number of published articles and review articles in industrial wastewater research for most productive Arab countries and non-Arab Middle Eastern countries

**Fig. 3:**
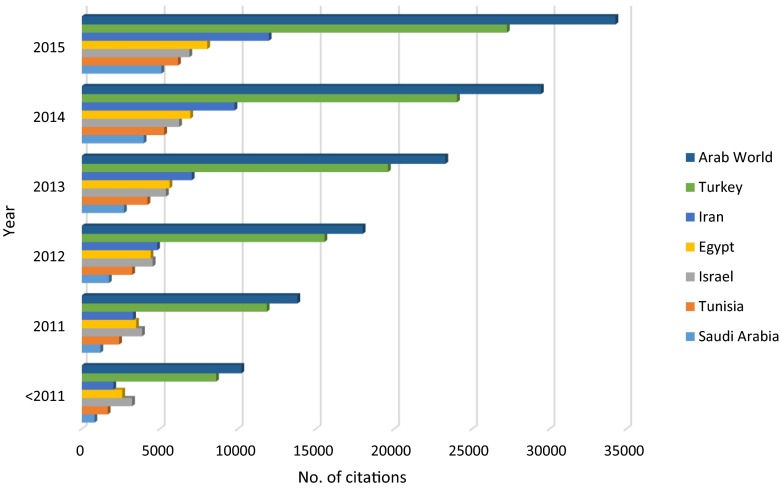
Developing of citations for published articles and review articles in industrial wastewater research for Arab world and most productive Arab countries and non-Arab Middle Eastern countries

Table 7 presents the results of a comparative analysis between the three most productive countries in the Arab world (Egypt, Saudi Arabia, and Tunisia) and three non-Arab Middle Eastern countries (Turkey, Israel, and Iran) with regard to number of published documents, citations, *h*-index, collaboration countries, and research output from collaboration. Figure 2 displays the evolution of scientific research in industrial wastewater in the three most productive countries from the Arab world (Egypt, Saudi Arabia, and Tunisia) and three non-Arab Middle Eastern countries (Turkey, Iran, and Israel). A comparison for the growth of rate of citations between the three most productive countries from the Arab world (Egypt, Saudi Arabia, and Tunisia) and three non-Arab Middle Eastern countries (Turkey, Iran, and Israel) is illustrated in Fig. 3.

**Table 7:** Qualitative and quantitative comparison between Arab world, three most productive countries—Arab world and three major Middle East countries

Region/country	Arab world	Egypt	Saudi Arabia	Tunisia	Turkey	Iran	Israel
Field							
No. of published documents	2,032	655	300	297	1,377	1,042	260
*h*-index	70	43	36	35	71	49	36
Total no. of citations	34,296	8,044	5,104	6,171	27,237	11,988	6,900
Mean of citations	16.88	12.28	17.01	20.78	19.78	11.5	26.54
Median of citations (Q1–Q3)	6 (1.0–16.0)	4 (1.0–14.0)	6.5 (2.0–16.0)	9 (3.0–20.0)	8 (2.0–21.0)	4 (1.0–12.0)	9 (4.0–22.0)
No. of collaboration countries	66	42	42	31	52	39	39
No. of documents from collaboration (%)	715 (35.2)	183 (27.9)	179 (59.7)	139 (46.8)	165 (12.0)	158 (15.2)	8,532.7
Most collaborated country (no. of documents (%))	France (239–11.8 %)	Saudi Arabia (55–8.4 %)	Egypt (55–18.3 %)	France (78–26.3 %)	USA (48–3.5 %)	USA (28–2.7 %)	USA (37–14.2 %)
Most used language (no. of documents (%))	English (1,957–96.3 %)	English (650–99.2 %)	English (299–99.7 %)	English (285–96.0 %)	English (1,334–96.9 %)	English (972–93.3 %)	English (259–99.6 %)
Used mother language (no. of documents (%))	Arabic (0.0–0 %)	Arabic (0.0–0 %)	Arabic (0.0–0 %)	Arabic (0.0–0 %)	Turkish (54–3.9 %)	Persian (75–7.2 %)	Hebrew (0.0–0 %)
Most productive institution (no. of documents (%))	National Research Centre, Egypt (169–8.32 %)	National Research Centre, Egypt (169–30.4 %)	King Abdulaziz University (75–25.0 %)	University of Sfax (72–24.2 %)	Istanbul Teknik Universitesi (158–11.5 %)	Islamic Azad University (203–19.5 %)	Hebrew University of Jerusalem (59–22.7 %)
Most used journal (no. of documents (%))	Desalination (126–6.2 %)	Desalination (26–4.0 %)	Desalination (12–4.0 %)	Desalination (20–6.7 %)	Journal of Chemical Technology and Biotechnology (94–6.8 %)	Journal of Environmental Studies (52–5.0 %)	Desalination (20–7.7 %)

## Discussion

This study is an attempt to measure the performance indicators related to the Arab world research productivity in industrial wastewater research. Our approach was to source publications and to gather systematic data from Scopus databases, employing the bibliometric techniques which are used frequently to examine the trends and the scientific output (Gunn et al. 2015) in many disciplines of science (Sweileh et al. 2015; Zheng et al. 2015; Zyoud et al. 2015; Zyoud and Fuchs-Hanusch 2015). Qualitative and quantitative parameters have been considered to measure and compare the performance of research activities originated from researches in the Arab world against other regions. The improving of the research activities and the scientific quality is a pre-condition to open up new fields of knowledge (Tijssen et al. 2002).

This study has addressed the issue of evaluating the quantity of industrial wastewater-based research in the Arab world by utilizing the total amounts of publications and the quality of research by utilizing the impact factors, *h*-index, and citation rates. The performance indicators, in terms of research productivity, showed that research activities in this field are insufficient or even ignored in some Arab countries. The research activities in industrial wastewater at the Arab world level have begun very modestly from the middle of seventieth of the twentieth century and developed gradually. The launch of these activities was too delayed when compared to the beginnings of research activities in this field at global level. It is to know that the first documented article in Scopus databases, related to industrial wastewater research at global level, was in 1931, whereas the first published article by researches from the Arab world was in 1976. In spite that time lags are observed, this launch was concurrent with the beginning of the real momentum in industrial wastewater research at global level which was trivial and sporadic before the sixteenth of the twentieth century. It was relatively in parallel with the start of research activities in this field in other major Middle East countries.

Despite the encouraging indicators of increasing research productivity, and the encouraging trends shown by citation indicators during the last period, there are large gaps in productivity between the developed and developing countries as pointed out by data announced by the Institute for Scientific Information (Langer et al. 2004). The Arab world is responsible for a meager 1.4 % of the scientific papers published worldwide (Boumedjout 2010). The increase in research activities in industrial wastewater from the Arab region could be associated with the general increase in research and publication activity (Benamer and Bakoush 2009). The contribution in global scientific research from the Arab world in industrial wastewater showed a figure of 3.6 %, which is better than the Arab world contribution in solid waste research that showed a figure of 2.35 % (Zyoud et al. 2015), and is much less than the Arab world contribution in desalination research, which amounted to 16.0 % (Zyoud and Fuchs-Hanusch 2015). This figure is also better than the results in medical research; Arab countries currently produce less than 1 % of citations in the world and contribute less than 0.5 % of papers appearing in the 200 leading medical journals (Maziak 2005). These findings were also proved by numerous bibliometric studies in medical research (Sweileh et al. 2015; Sweileh et al. 2014). Diabetes mellitus research output from the Middle Eastern Arab countries showed a figure of 0.75 % of the total documents produced globally (Sweileh et al. 2014). The justification of the reasonably good figure in industrial wastewater research is explained by the interactions between industrial wastewater research and desalination research. It is proved that Arab countries excel in desalination research (Masood 2002).

The prevalent used language in industrial wastewater research by researchers from the Arab world was English. The English language is widely accepted as the international language of science and technology. It is the current global lingua franca of international scientific publications (Vanbaelen and Harrison 2015). Arab researchers tend to publish in English language to gain international credibility (Atwell et al. 2009). Research papers written in Arabic have restricted circulation as pointed out by the initiative of the Arab League Educational, Social, and Cultural Organization (ALESCO) to run workshops where leading-edge research is reported in Arabic, but this was anticipated making the proceedings inaccessible to the worldwide research community (Atwell et al. 2009). In Turkey and Iran, there is increase in using the mother languages in their research activities. The rapidly growing of using these languages could be justified by the increase in the number of journals from these two countries indexed in Scopus (Najari and Yousefvand 2013).

Egypt, Saudi Arabia, and Tunisia markedly showed higher rates of contributions in industrial wastewater research. In the case of Egypt, it has the most extensive research structure in the Arab world in terms of research and development units and has traditional strengths in chemistry and engineering research (Koenig 2007). Egypt is the second on its continent (after South Africa) in scientific production (Waast and Rossi 2010). The population size of Egypt is a vital factor among other factors in increasing the scientific research productivity since it depends on population, socioeconomic, or overall scientific activity of the country (Miró et al. 2009). The interests in industrial wastewater research in Egypt stem partially from developing numerous management programs for industrial wastewater. These management programs have been built to eliminate the risks imposed by industrial wastewater after constructing new industrial estates in the desert and to protect the ecosystem of the Nile river and delta from further degradation (El-Gohary et al. 2002). The heavy use of water in food industry made Egyptian researchers focus their efforts on developing new technologies to treat the wastewater effluents. Another important subject of research in Egypt is the agricultural wastewater treatment (Barceló and Petrovic 2011). Three institutions in Egypt claim top positions in the list of top ten institutions at the Arab world level. The National Research Center (NRC), which occupies the top position in the list, is the largest research center in the Middle East for science and technology. The center serves the national, regional, and international cooperation including technology transfer and membrane technology in water treatment (Lorenzo 2010).

Saudi Arabia is the most collaborated country with Egypt in industrial wastewater research. They have a strong research partnership. The annual joint research between the two countries has risen tenfold in the past decade and is accelerating (Adams 2012). The USA is the biggest partner outside the region for both countries (Adams 2012). This conclusion has also been demonstrated in this study where the USA has assumed the second position in collaboration with Egypt and Saudi Arabia. The study showed that 43 (6.6 %) and 31 (10.3 %) documents out of the total documents published by Egypt and Saudi Arabia, respectively, were in collaboration with the USA.

The oil refining and natural gas processing industries, as in Saudi Arabia, are responsible for approximately 38 % of the annual industrial water withdrawals (Kajenthira et al. 2012). There are growing interests in industry sectors in Saudi Arabia to treat and reuse the industrial wastewater (Al-Ghasham et al. 2005). This will reduce their water demands from desalination plants and municipal sources and consequently reduce the costs and will save energy (Wichelns et al. 2015). With regard to scientific research and development, Saudi Arabia is progressing and has plans to increase funding for research to the amount of 2 % of the total gross domestic product by 2015 (Alshayea 2013). The strategy of Saudi Arabia is setting up a base of science by localizing the best foreign capabilities and innovative research and development firms in knowledge villages (Waast and Rossi 2010). King Abdul-Aziz University of Saudi Arabia claims the second position in the list of most prolific institutions in the Arab world in industrial wastewater research. It is the home of center of excellence in desalination technology. These findings reflect the efforts of Saudi Arabia for the advancement of scientific research and the special consideration which has been given to scientific research in order to promote scientific innovation, as well as to develop universities and research centers (Alshayea 2013).

Tunisia is considered among the first countries in the Mediterranean region that established and implemented an integrated wastewater reuse policy (Kellis et al. 2013). It is recognized, along with Israel, as a leader in the area of wastewater reclamation and reuse (Shetty 2004). The classification of Tunisia as one of the least developed countries endowed with water resources in the Mediterranean basin is the driving force behind the intensive investment in industrial wastewater treatment research (Jemli et al. 2015). Tunisia has a pioneer institution which is one of the leading institutions in the field of environmental biotechnology in the African, Middle East, and North African regions. The Centre of Biotechnology of Sfax is working on developing new concepts for bioremediation of waste and wastewater, etc. The Sfax Laboratory of Environmental Bioprocesses concentrates its research on studying the biological system involved in aerobic and anaerobic degradation of municipal and industrial pollutants (Lorenzo 2010). The PROMEMBRANE project, which was funded by the European Union, with a primary objective to support and focus the current research and development activities on membrane technology for water treatment in the Mediterranean region, has affected positively in increasing research activities in Tunisia (Lorenzo 2010).

The collaboration patterns between Arab world and non-Arab countries demonstrated high rates of collaboration with Western Europe region, mainly France. This could be explained by the active participation of the three Maghreb countries, Tunisia, Algeria, and Morocco, which contributed in 30 % of the total research output from the Arab world. Their collaboration with France resulted in 215 (32.0 %) documents out of their research output. France was noted to be heavily associated with its former colonies in external research collaboration. The results of collaboration between France and Maghreb countries were compatible to the results of analysis conducted by Harford (2015) to examine the optimal collaboration in cancer control efforts across the Africa continent (Harford 2015). The USA took the second place in collaboration with the Arab world. The USA sustains as a major contributor in scientific collaboration due to its large productivity in scientific research (Gazni et al. 2012). The benefits of international collaboration are manifested in its powerful in bringing complementary expertise together to pursue and achieve higher-impact science research (Havemann et al. 2006). Additional benefits are in reducing the costs and saving the efficiency by reducing the duplication of equipment and expertise (Toope et al. 2012).

The screening of the Arab world performance in comparison with three non-Arab Middle Eastern countries (Turkey, Iran, and Israel) at country level shows a gap especially with comparison to the performance of Turkey. The performance of Tunisia, in spite of its limited resources and the size of its population, was unique. The adoption of European Union standards for water, wastewater, and solid waste management in Turkey, associated with the high rates of industrial wastewater (75 %) discharged without treatment, motivated researchers to study and examine new treatment technologies for highly polluted industrial wastewater (Malato et al. 2011).

The analysis provides an integrated perspective and interesting insights on the figuration and developing of industrial wastewater research, figures of research activity distribution, qualitative aspects, and collaboration trends. In bibliometric analyses, several limitations could be raised. One of these limitations is the employing of Scopus databases only. This study cannot take into consideration all literatures published in other databases which might have contribution to industrial wastewater research in the Arab world.

## Conclusions

A remarkable evidence of commitment to increase research activities in industrial wastewater research has been noticed in the Arab world. A dramatic boost in the number of published documents by researchers from the Arab world took place in the last 10 years, and the quality indicators of these researches are promising in the field of industrial wastewater. Egypt, Saudi Arabia, and Tunisia are the leading Arab countries in this regard. Despite the optimistic outlook, there are still gaps between the Arab countries and other countries in the same region and at global level. Research institutions in Arab world should strengthen collaboration networks with their counterparts in developed countries to promote research activities. The governments should invest in industrial wastewater research as a requirement and not a choice. These investments are necessary to eliminate the potential risks of industrial wastewaters and to optimize the use of water resources. This paper serves as a benchmark which makes comparisons among the Arab world countries and other major countries in the region. The applied bibliometric methodology is applicable to other subjects in environmental realm.
